# From Disease Description and Gene Discovery to Functional Cell Pathway: A Decade-Long Journey for TMCO1

**DOI:** 10.3389/fgene.2021.652400

**Published:** 2021-05-20

**Authors:** Helen Batchelor-Regan, Baozhong Xin, Aimin Zhou, Heng Wang

**Affiliations:** ^1^DDC Clinic Center for Special Needs Children, Middlefield, OH, United States; ^2^Clinical Genetics Department, Birmingham Women’s and Children’s NHS Foundation Trust, Birmingham, United Kingdom; ^3^Department of Chemistry, Center for Gene Regulation in Health and Diseases, Cleveland State University, Cleveland, OH, United States; ^4^Department of Pediatrics, Rainbow Babies and Children’s Hospital, Cleveland, OH, United States; ^5^Department of Molecular Cardiology, Cleveland Clinic Foundation, Cleveland, OH, United States

**Keywords:** TMCO1, CFTD, TMCO1 defect syndrome, CLAC channel, calcium, cerebrofaciothoracic dysplasia

## Abstract

A decade has passed since transmembrane coiled-coil domains 1 (TMCO1) defect syndrome was identified in 11 undiagnosed patients within the Old Order Amish of Northeastern Ohio—a disorder characterized by a distinctive craniofacial dysmorphism, skeletal anomalies and global developmental delay. Twenty seven patients, from diverse ethnic groups, have been reported with pathogenic TMCO1 variants now recognized to cause cerebrofaciothoracic dysplasia (CFTD). The implication of previously uncharacterized TMCO1 within disease has instigated a 10-year journey to understand the function of TMCO1 protein in Ca^2+^ homeostasis. TMCO1 is an ER Ca^2+^ leak channel which facilitates Ca^2+^ leak upon ER “overload” through the novel Ca^2+^ load activated Ca^2+^ mechanism. This mini-review brings together the clinical and scientific advances made since the discovery of TMCO1 deficiency in disease, including broadened phenotype, understanding of pathophysiology, and implications to patient management of TMCO1 defect syndrome.

## Introduction

Beginning in the 1960s, inherited disorders among Old Order Amish communities were the subject of exceptional observational studies (McKusick, 1978) and many conditions still remain subjects of extensive research. During the last two decades, new technologies and methodologies in genomic medicine have brought dramatic progress on the studies of genetic disorders in isolated populations. We share here our decade-long journey to understanding a novel rare genetic disorder: Transmembrane and coiled-coil domains 1 (TMCO1) defect syndrome; from phenotype description to functional studies in both cell and animal models, and application of learnings back to patient management. This endeavor exemplifies how translational genomic medicine can benefit affected patients and families both within Plain communities and the broader international rare disease community, and demonstrates how a rare genetic disorder may help us to understand common and essential biological pathways in human cells.

## From Patients to Gene Discovery: Identification of TMCO1 Defect Syndrome in the Ohio Amish

The Amish communities of North America represent unique populations due to their closed communities and small founding populations. They descend from Swiss Anabaptist immigrants who came to the New World in the early eighteenth century. There is increased incidence of rare recessive disorders in Amish populations, caused by founder effects. The majority of affected individuals are homozygous for their respective pathogenic variant, which resides within a relatively large homozygous haplotype block. Biological and social aspects of the Amish communities make them ideal participants for studies in population genetics and genomic medicine.

High density single-nucleotide polymorphism (SNP) microarray genotyping has been successfully used in linkage analysis and genome-wide homozygosity mapping for undiagnosed recessive conditions within the Amish communities. Using SNP array genotyping data, a disease locus can be mapped to one specific chromosome region with a few affected patients. This historic breakthrough was particularly significant in identifying genetic causes of rare conditions and gene discovery before the era of next-generation sequencing (Puffenberger et al., 2004).

In 2010, we identified a novel autosomal recessive condition in a group of 11 undiagnosed patients within the Old Order Amish of Northeastern Ohio with similar clinical phenotypes (Xin et al., 2010). All were born with normal birth weight but hypotonia and poor feeding. The shared phenotype was characterized by distinctive craniofacial dysmorphism, skeletal anomalies, and global developmental delay. The typical craniofacial dysmorphism included brachycephaly, highly arched bushy eyebrows, synophrys, long eyelashes, low-set ears, microdontism of primary teeth, and generalized gingival hyperplasia. Medical photographs detailing facial features can be found within the original article (Xin et al., 2010). Skeletal anomalies included Sprengel deformity of scapula, fusion of spine, rib abnormalities, pectus excavatum, and pes planus. Neurological examination revealed depressed deep tendon reflexes, unstable gait, and, in some older patients, intention tremor. Imaging studies demonstrated mild prominence of ventricles. Sluggish speech with a loud, hoarse voice was found in all verbal patients (6/11 individuals). Anatomical abnormalities of the genitourinary system were noted, including renal agenesis, hydronephrosis, vesicoureteral reflux, hypoplastic labia minora, hydrocele, and undescended testes. Sudden death occurred in two patients in their late 20s.

Genome-wide SNP array genotyping and autozygosity mapping identified a single shared homozygosity region of 3.3 Mb in all patients at chromosome 1q23.3-q24.1 containing 23 candidate genes (Xin et al., 2010). DNA sequencing revealed a previously unreported homozygous 2-bp deletion in the TMCO1 gene (c.139_140delAG); this introduces a premature stop codon and results in a severely truncated protein (p.Ser47Ter). It co-segregated consistently with disease phenotype (Xin et al., 2010). Our report, for the first time, showed TMCO1 gene deficiency being associated with an adverse phenotype in humans; this condition was therefore designated as TMCO1 defect syndrome.

## Understanding the Function of the TMCO1 Protein

TMCO1 is a highly conserved 188 amino acid cation channel with a predicted molecular mass of 21.2 kD (Iwamuro et al., 1999). TMCO1 is ubiquitously expressed in human adult and fetal tissues, with high expression noted in heart, liver, thymus, prostate, testis, and kidney (Iwamuro et al., 1999; Xin et al., 2010; Zhang et al., 2010). Sequence analysis predicts a structure with two transmembrane domains (amino acids 9–31 and 91–109), and a further hydrophobic segment (amino acids 143–154) (Iwamuro et al., 1999; Wang et al., 2016), with both the N- and C-termini located in the cytosol (Wang et al., 2016). Nuclear magnetic resonance studies have conversely suggested three transmembrane domains. Native TMCO1 channel structure has remained elusive due to difficulties in expressing and isolating TMCO1 for crystallography studies (Zhang et al., 2021). Multiple studies have indicated that TMCO1 is localized to the endoplasmic reticulum (ER) membrane and mitochondria (Iwamuro et al., 1999; Zhang et al., 2010; Wang et al., 2016). TMCO1 is readily diffusible throughout the ER membrane. Assessment of channel dynamics through inside-out patch clamping defined TMCO1 as a Ca^2+^-selective cation channel. The negative charge of pore-lining residue D140 is required for Ca^2+^ permeation, with a charge-neutralizing mutation abrogating Ca^2+^ extrusion from the ER. No currents are detectable through the TMCO1 (p.Ser47Ter) mutant protein (Wang et al., 2016).

The ER is a critical intracellular Ca^2+^ store, with store load being finely balanced. Ca^2+^ homeostasis is regulated through a balance of Ca^2+^ uptake via sarcoendoplasmic reticulum Ca^2+^ ATPase (SERCA) pumps, and release through Ca^2+^ leak channels. ER Ca^2+^ leak is currently not well defined.

TMCO1 has been established as an ER Ca^2+^ leak channel (Wang et al., 2016). Knockdown (KD) of TMCO1 resulted in ER Ca^2+^ store overload, indicating loss of endogenous Ca^2+^ leak. Store depletion resulted in a two-fold increase in total Ca^2+^ released from ER stores in TMCO1 KD cells. Measurement of ER Ca^2+^ load showed significant increase in basal ER Ca^2+^ concentration (Wang et al., 2016). TMCO1 Amish variant (p.Ser47Ter) expression did not rescue Ca^2+^ leak, indicating abnormal Ca^2+^ handling as a mechanism for the disease phenotype. TMCO1-knockout (KO) mouse showed severe ER Ca^2+^ mishandling within osteoblasts and granulosa cells, although no overload was observed in germinal vesicles (Wang et al., 2016; Sun et al., 2018). This suggests the importance of other leak channels in different cell types.

**FIGURE 1:**
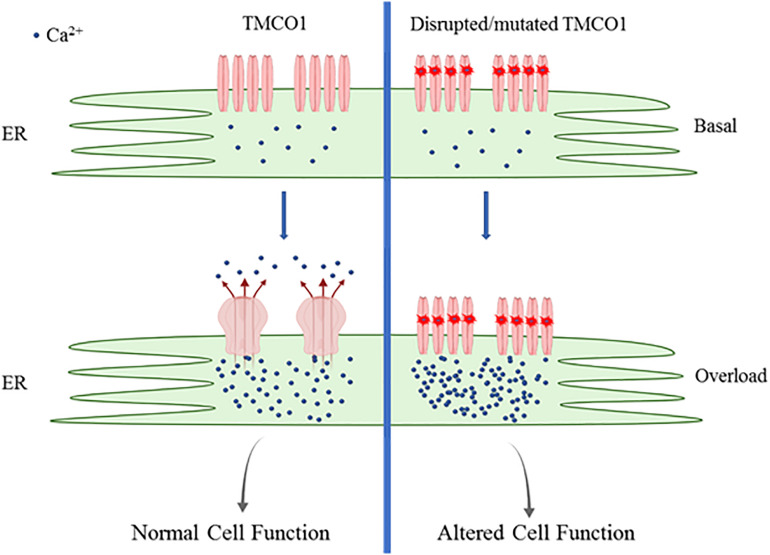
Schematic of TMC01 function. TMCO1 exists as monomers and dimers during basal ER Ca^2+^ load. ER Ca^2+^ reaching overload results in oligomerization to form active TMCO1 tetrameric channels, facilitating Ca^2+^ release. Channels undergo rapid disassembly once basal Ca^2+^ levels are restored. Mutated TMCO1 is unable to assemble to form active channels resulting in persistent ER Ca^2+^ overload.

TMCO1 functions in a novel Ca^2+^-load activated Ca^2+^ (CLAC) mechanism. Increases of ER Ca^2+^ concentrations to “overload” levels result in endogenous TMCO1 monomers and dimers undergoing oligomerization to form homotetramers. Tetrameric TMCO1 channel facilitates active Ca^2+^ leak from the ER to reduce Ca^2+^ load to resting ER concentrations as illustrated in Figure 1 (Wang et al., 2016). The Ca^2+^-sensing mechanism of TMCO1 has not yet been investigated. Further studies are required to determine if TMCO1 can self-detect ER Ca^2+^ load or whether a secondary Ca^2+^-sensing protein is required.

ER Ca^2+^ homeostasis is important in maintaining ER protein synthesis and membrane lipid biogenesis. The activity of many ER chaperones and foldases utilize Ca^2+^as a cofactor, binding Ca^2+^ with low affinity (reviewed in Carreras-Sureda et al., 2018). ER Ca^2+^ stores are also required for both store-operated Ca^2+^ entry (SOCE) and Ca^2+^-induced Ca^2+^ release (CICR) signaling. SOCE is an important signaling mechanism in non-excitable cells: facilitated by the Ca^2+^-release activated Ca^2+^ (CRAC) channel. CICR is thought to play an important role in Ca^2+^ signaling in cardiac smooth muscle.

Understanding the effect of disturbed ER Ca^2+^ homeostasis on downstream functions is critical to understand TMCO1 deficiency pathophysiology. TMCO1-KO mouse displays a disease phenotype consistent with TMCO1 defect syndrome, including delayed osteogenesis, cognitive disability, and unstable gait (Wang et al., 2016). Female TMCO1-KO mice additionally show impaired follicle development, subfertility, and premature ovarian failure (POF) (Sun et al., 2018).

ER stress has been identified in TMCO1 KD and KO cell lines and animal models (Sun et al., 2018; Wang et al., 2019). ER Ca^2+^ overload due to TMCO1 deficiency results in increased intracellular and mitochondrial reactive oxygen species (ROS) and ER stress-mediated apoptosis through the IRE1 pathway (Sun et al., 2018). ER stress initiates the unfolded protein response (UPR) and ER-associated degradation (ERAD) (Carreras-Sureda et al., 2018). Alterations in protein expression profiles have been observed in TMCO1 deficient cells. Diacylglycerolacyltransferase (DGAT)-2 is required for the final and rate-limiting step of triglyceride production. DGAT-2 protein levels, but not mRNA levels, are reduced in TMCO1 KD cells due to enhanced ERAD (Wang et al., 2019). This results in impaired fatty acid (FA) esterification and reduced intracellular lipid droplets (LD) during ER Ca^2+^ overload. Store depletion results in LD increase to normal levels.

LDs play a role in the maintenance of mitochondria and prevention of lipotoxicity by reducing free cytoplasmic FAs. A reduction in mitochondrial volume due to increased mitophagy was observed in TMCO1 KO and KD cells. Mitochondria showed poor morphology including abnormal cristae morphology and presence of large vacuoles in TMCO1 KO, and mitochondrial respiration was found to be significantly reduced (Wang et al., 2019). These findings demonstrate possible mechanisms by which TMCO1 deficiency results in pathophysiology. Further research is required to understand the impact of the loss of TMCO1 through ER stress and implications on Ca^2+^ signaling.

## Developing Our Clinical Understanding of Disease Genotype and Phenotype

Four years after the publication of the TMCO1 defect syndrome, Alanay et al. identified a pathogenic TMCO1 variant within four Turkish families diagnosed with cerebrofaciothoracic dysplasia (CFTD) (Alanay et al., 2014). They proposed that TMCO1 defect syndrome was part of the CFTD spectrum of disorders, originally identified by Pascual-Castroviejo (Pascual-Castroviejo et al., 1975). This is supported in subsequent literature. TMCO1 and the 1q24 region were excluded within one family, indicating that CFTD may be a heterogeneous disorder (Alanay et al., 2014). Furthermore, the molecular etiology of individuals with a clinical diagnosis of CFTD, prior to the identification of TMCO1, has not been reported in literature. “TMCO1 defect syndrome” will therefore be used within this review to define any individual with an identified pathogenic variant in TMCO1 causing a clinical presentation of CFTD.

Twenty-seven patients, from diverse ethnic backgrounds, are reported in literature as having TMCO1 defect syndrome. Five further pathogenic variants have been identified. All identified variants result in a premature termination codon (PTC), including a splicing variant which results in exon five skipping and early termination in exon six. Nonsense- mediated mRNA decay has been proposed for 4/5 variants, with 1/5 variants predicted to produce a non-functional protein product (Caglayan et al., 2013; Alanay et al., 2014; Pehlivan et al., 2014; Tender and Ferreira, 2018; Michael Yates et al., 2019; Sharkia et al., 2019). It is therefore likely that the complete loss of function of TMCO1 is critical in the pathophysiology of TMCO1 defect syndrome.

An additional five variants have been reported as pathogenic on ClinVar but not reported in literature (TMCO1[gene], 2020). 4/5 variants also result in early termination, consistent with variants reported in literature. The 5th variant results in a frameshift, which is also predicted to produce a truncated protein. These additional variants indicate a larger patient cohort than literature suggests.

The increase in patient cohort size has improved understanding of both disease genotype and phenotype. Full reported clinical manifestation information is available within Supplementary Table 1. Additional facial features include a wide anterior fontanelle, narrow forehead, posteriorly rotated ears, and jaw anomalies such as prognathism and micrognathia. Cerebral imaging findings have been broadened from the ventricle abnormalities initially described in TMCO1 defect syndrome. Corpus callosum abnormalities have been reported in 57% of new TMCO1 defect syndrome cases. Further anomalies within the cerebellar and frontotemporal lobe have been reported. Epilepsy has been seen in three cases. Variance in stature is marked, with 55% individuals > 95th or < 5th centile where reported in literature. Vertrabral anomalies were described in 80% of new cases.

Key features initially reported by Xin et al have been reinforced by these additional cases. Most notable craniofacial features include low hairline, low-set ears, highly arched bushy eyebrows with synophrys, orbital hypertelorism, and brachycephaly with a flat face. Skeletal anomalies affecting the spine, ribs and scapula are common, including rib fusions and spine fusions. Severe intellectual difficulties have been reported in all cases. Developmental milestones have not been consistently addressed in clinical reports; however, 50% of individuals have been reported as non-verbal, with verbal individuals reported to have delayed first words, speech defects, and a small limited vocabulary. Developmental motor milestones are also delayed, with some individuals unable to walk and significant delay in age of independent sitting reported. Behavioral problems, such as anxiety and self-mutilation, have been described (Xin et al., 2010; Caglayan et al., 2013; Alanay et al., 2014; Tender and Ferreira, 2018; Michael Yates et al., 2019; Sharkia et al., 2019).

## From Bench Work and Disease Description to Practice: Clinical Management of TMCO1 Defect Syndrome

TMCO1 defect syndrome phenotype has been well described; however, disease development over time has been scarcely reported in literature. Further case reports of individuals may provide insight into disease progression and the effectiveness of treatments and interventions. With that in mind, this mini-review recommends clinical management based on current evidence.

Assessment of skeletal and neurological abnormalities should be completed. Speech and language therapy and physical therapy may provide benefit to individuals. Genital urinary tract abnormalities have been identified in 37% individuals, including kidney anomalies in four individuals. Assessment by a renal team is therefore advisable.

Cardiac anomalies have been reported in three individuals, including a narrow patent ductus arteriosus (PDA) present at age 4, hypertrophic left ventricle at 9 months, a systolic heart murmur at birth (Alanay et al., 2014; Michael Yates et al., 2019). A small PDA has also been reported in clinical diagnosis of CFTD (Cilliers et al., 2007). Cardiac abnormalities were reported in a fetus from a TMCO1 defect syndrome family (Pehlivan et al., 2014). Sudden death has additionally been reported (Xin et al., 2010). Cardiology review and further monitoring is therefore recommended for TMCO1 defect syndrome patients.

A history of spontaneous abortions has been reported in families affected by TMCO1 defect syndrome (Xin et al., 2010; Caglayan et al., 2013; Pehlivan et al., 2014). TMCO1 deficiency in products of conception has not been confirmed in any study; however, multiple congenital abnormalities consistent with TMCO1 defect syndrome have been reported in multiple fetuses within known TMCO1 defect syndrome families (Alanay et al., 2014; Pehlivan et al., 2014). The TMCO1 KO mouse model is reported to have reduced KO pup viability at 8.9% (Wang et al., 2016). Counseling families about the increased risk of miscarriage in affected fetuses is therefore advisable.

TMCO1 has been linked with other conditions. Primary open-angle glaucoma (POAG) is a heritable common cause of blindness, with a single modifiable risk factor of intraocular pressure (IOP), which is also highly heritable (Coleman and Miglior, 2008). GWAS studies have identified numerous risk loci associated with TMCO1 in both POAG and IOP (Burdon et al., 2011; van Koolwijk et al., 2012; Choquet et al., 2018; MacGregor et al., 2018). Individuals homozygous for the rs4656461 TMCO1 risk allele have been found to develop glaucoma 4–5 years earlier than non-carriers in families with a strong family history (Sharma et al., 2012); however, none of the reported TMCO1-associated risk alleles are located in coding regions of the TMCO1 gene. It has been proposed that variants within the regulatory region of TMCO1 confer increased risk through unknown mechanisms (Burdon et al., 2011; Sharma et al., 2012). No changes in TMCO1 protein expression have been demonstrated for those risk alleles and POAG and IOP have not been reported in TMCO1-deficient patients. Nevertheless, it is important to monitor IOP in these patients until understanding of TMCO1 involvement is further clarified.

## Future Research

It is now understood that TMCO1 is a CLAC channel with a role in maintaining ER Ca^2+^ homeostasis, although how the deficiency results in the clinical presentation of CFTD is not fully understood. Improving our understanding of the pathophysiology of CFTD will be significant in developing potential treatments to alleviate symptoms.

Further research is required into the regulation of TMCO1-dependent Ca^2+^ leak, including TMCO1 Ca^2+^ sensing mechanism and the impact of store overload on store dependent Ca^2+^ signaling pathways. Ca^2+^ signaling has been shown to be altered due to TMCO1 deficiency but has not been investigated in depth (Wang et al., 2016; Sun et al., 2018). Aberrant ER-dependent Ca^2+^ signaling is associated with disease. Deficiencies of CRAC channel components, Orai and STIM, are well known in their link to innate and acquired immunity. They have also been associated with congenital myopathies, anhydrosis, dental defects, pancreatitis, and kidney issues (McCarl et al., 2009; Feske et al., 2010; reviewed in Putney et al., 2017). Some phenotypic overlay between TMCO1 defect syndrome and other disorders associated with CRAC channel components is notable and further TMCO1 defect studies may also support wider understanding of the pathology of CRAC channel defect.

TMCO1 is reported to be ubiquitously expressed in both embryonic and adult tissues (Iwamuro et al., 1999; Xin et al., 2010; Zhang et al., 2010). TMCO1 defect syndrome shows distinct skeletal anomalies and severe developmental delay consistent with loss of TMCO1 impacting early embryonic tissue development. Differential expression of TMCO1 in embryos has not been quantified (Xin et al., 2010). Further study of embryonic TMCO1 expression and examination of fetal development in the TMCO1 KO mouse model may improve understanding of phenotype development.

Loss of ER Ca^2+^ homeostasis has been shown to increase ER stress, resulting in UPR, ERAD, and IRE1-dependent apoptosis and is clearly linked to the pathophysiology of TMCO1 defect syndrome (Sun et al., 2018; Wang et al., 2019). This is an area requiring further research, particularly in understanding the impact on key tissues, including neurons and osteoblasts. Although aberrant Ca^2+^ signaling may contribute to TMCO1 defect syndrome phenotype, it seems likely that perturbations in other ER processes through ER stress mechanisms play a more principle role. Further translational research with the emphasis on the normalization of CLAC channel and ER Ca^2+^ homeostasis as potential therapeutic approaches is certainly our ultimate hope for the affected patients and future research direction.

## Summary

A decade-long journey has defined TMCO1 defect syndrome as a form of CFTD, and has instigated research in to the function of TMCO1 protein. The characterization of TMCO1 function as a novel CLAC channel, with a role in maintaining ER Ca^2+^ homeostasis, has significantly improved our understanding of the pathophysiology of CFTD. This is an example of the impact of rapidly evolving genomic technologies on gene discovery as well as the consequent improvement of patient management. It also demonstrates how rare disease studies may help us understand essential functional pathways in cell biology.

## Author Contributions

HW initiated the concept and outline. HB-R drafted the manuscript. All authors contributed ideas, edited, and approved the final version.

## Conflict of Interest

The authors declare that the research was conducted in the absence of any commercial or financial relationships that could be construed as a potential conflict of interest.
