# Supplementary material for: From Disease Description and Gene Discovery to Functional Cell Pathway: A Decade-Long Journey for TMCO1
Source: Front Genet. 2021 May 20;12:652400. doi: 10.3389/fgene.2021.652400 (PMC8172970; doi:10.3389/fgene.2021.652400)
Supplement: Supplementary file 1 [file Table_1.DOCX]

|  |  | ***Paper*** | ***Xin et al, 2010*** | ***Cagyalan et al, 2013*** | ***Alanay et al, 2014*** | | ***Pehlivan et al, 2014*** | ***Tender and Ferreira, 2018*** | ***Michael Yates et al, 2019*** | | ***Sharkia et al, 2019*** | ***Totals*** |
| --- | --- | --- | --- | --- | --- | --- | --- | --- | --- | --- | --- | --- |
|  |  | *Ethnicity* | *Old Order Ohio Amish* | *US non-Amish* | *Turkish* | | *Turkish* | *Guate-malan* | *Northwest Pakistani* | *Scottish* | *Arab* |  |
|  |  |  |  |  | *Fetus* | *Patient* |  |  |  |  |  |  |
| Prenatal and neonatal | Prenatal | Polyhydroamnios | 4/11 | - | 0/2 | 1/4 | 1/1 | 0/2 | - | - | 2/2 | 7/22 (32%) |
|  |  | Decreased foetal movements | 4/11 | - | 0/1 | 0/3 | - | - | - | - | 2/2 | 6/17 (35%) |
|  |  | Abnormal ultrasound findings | - | - | 2/2 | 2/4 | - | - | 1/3 | 1/1 | - | 6/10 (60%) |
|  | Neonatal | Hypotonia | 11/11 | 1/1 | - | - | 1/1 | 2/2 | 0/3 | 0/1 | 2/2 | 17/21 (81%) |
|  |  | Poor feeding | 11/11 | 1/1 | - | 4/4 | - | - | 0/3 | 0/1 | 2/2 | 18/22 (82%) |
|  |  | Hypothyroidism | - | 1/1 | - | - | - | - | - | - | - | 1/1 (100%) |
|  |  | High birth weight/large for gestational age | - | - | 1/1 | 2/4 | 1/1 | - | 3/3 | 0/1 | 0/2 | 7/12 (58%) |
| Cranio-facial | Cranio | Macrocephalic appearance | 9/10 | 1/1 | 1/1 | 0/4 | 1/1 | - | 1/3 | 0/1 | 0/2 | 13/23 (57%) |
|  |  | Microcephalic appearance | - | - | - | 2/4 | - | - | 0/3 | 0/1 | - | 2/8 (25%) |
|  |  | Brachycephaly and flat face | 11/11 | - | 2/2 | 4/4 | 1/1 | 2/2 | 0/3 | 1/1 | 2/2 | 23/26 (88%) |
|  |  | Craniosynostasis | 2/11 | 0/1 | - | 1/2 | - | - | - | - | 0/2 | 3/16 (19%) |
|  | Facial | Wide anterior fontanelle | - | - | 2/2 | 1/1 | - | - | - | - | - | 3/3 (100%) |
|  |  | Narrow forehead | - | - | 2/2 | 3/4 | 1/1 | - | - | - | 2/2 | 8/9 (89%) |
|  |  | Frontal bossing | - | - | - | - | 1/1 | - | - | - | 1/2 | 2/3 (67%) |
| Cranio-facial | Facial | Wide nasal bridge | 11/11 | - | 2/2 | 4/4 | - | - | 0/3 | 1/1 | 2/2 | 20/23 (87%) |
|  |  | Short nose | 11/11 | - | 2/2 | 4/4 | 1/1 | 0/2 | - | - | 0/2 | 18/22 (82%) |
|  |  | Anteverted nares/ hypoplastic ala nasi | 11/11 | 1/1 | 2/2 | 4/4 | - | - | - | - | 0/2 | 16/18 (89%) |
|  |  | Broad nose tip | - | - | - | - | 1/1 | - | - | - | 0/2 | 1/3 (33%) |
|  |  | Long philtrum | - | - | - | - | 1/1 | - | - | - | - | 1/1 (100%) |
|  | Hair | Low hairline | 11/11 | 1/1 | 2/2 | 3/3 | 1/1 | 2/2 | - | - | 2/2 | 22/22 (100%) |
|  |  | Frontal upsweep | - | - | - | 3/3 | - | - | 0/3 | 1/1 | 2/2 | 6/9 (67%) |
|  | Ears | Low set ears | 11/11 | 1/1 | 2/2 | 4/4 | 1/1 | 2/2 | 3/3 | 1/1 | 2/2 | 27/27 (100%) |
|  |  | Posteriorly rotated ears | - | - | 2/2 | 4/4 | 1/1 | - | - | 1/1 | 0/2 | 8/10 (80%) |
|  | Eyes | Highly arched bushy eyebrows | 11/11 | - | 2/2 | 4/4 | 1/1 | 2/2 | 2/3 | 1/1 | 2/2 | 25/26 (96%) |
|  |  | Synophrys | 11/11 | 1/1 | 2/2 | 4/4 | 1/1 | 2/2 | 3/3 | 0/1 | 2/2 | 26/27 (96%) |
|  |  | Orbital hypertelorism | 11/11 | 1/1 | 2/2 | 3/4 | 1/1 | 2/2 | 2/3 | 1/1 | 2/2 | 23/25 (92%) |
|  |  | Epicanthal fold | - | - | 1/2 | 3/4 | 1/1 | - | 0/3 | 1/1 | 2/2 | 8/13 (62%) |
|  |  | Up/down slanting palpebral fissures | - | - | - | - | 1/1 | - | 1/3 | - | 2/2 | 4/6 (67%) |
|  |  | Long eyelashes | 11/11 | - | - | 4/4 | 1/1 | - | 0/3 | 1/1 | 2/2 | 19/22 (86%) |
| Oral and dental          Oral and Dental | Oral | High arched palate | 11/11 | 1/1 | - | - | 1/1 | - | 1/3 | - | 2/2 | 16/18 (89%) |
|  |  | Cleft lip and palate | 3/11 | - | 1/2 | 4/4 | - | 1/2 | 0/3 | 0/1 | 1/2 | 10/25 (40%) |
|  |  | Macroglossia | - | - | - | - | - | - | 1/3 | - | - | 1/3 (33%) |
|  |  | Everted lower lip | - | - | - | - | - | - | 1/3 | - | - | 1/3 (33%) |
|  | Dental | Microdontism of primary teeth | 9/9 | - | - | 3/3 | - | 2/2 | 1/3 | 0/1 | 2/2 | 17/20 (85%) |
|  | Dental | Generalised gingival hyperplasia | 8/8 | - | - | 2/2 | - | 2/2 | - | - | 2/2 | 14/14 (100%) |
|  | Jaw | Prognathism | - | 1/1 | - | - | - | - | - | - | - | 1/1 (100%) |
|  |  | Micrognathia | - | - | 2/2 | 4/4 | 1/1 | - | - | - | - | 7/7 (100%) |
| Skeletal | Neck and shoulders | Short neck | 6/11 | 1/1 | 2/2 | 4/4 | 1/1 | 2/2 | 1/3 | - | 0/2 | 17/26 (65%) |
|  |  | Scapula anomalies (including Sprengel deformity) | 8/10 | 1/1 | 0/1 | 2/4 | - | - | 1/3 | 0/1 | 2/2 | 14/22 (64%) |
|  |  | Narrow shoulders | - | - | - | - | 1/1 | - |  | - | - | 1/1 (100%) |
|  | Spinal | Vertrabral anomalies | - | - | 2/2 | 4/4 | 1/1 | 1/2 | 2/3 | 0/1 | 2/2 | 12/15 (80%) |
|  |  | Scoliosis | 7/11 | 1/1 | 0/2 | 2/4 | - | - | 2/3 | - | 2/2 | 14/23 (61%) |
|  |  | Fusion of spine | 6/11 | 0/1 | - | - | - | - | 1/3 | - | - | 7/15 (47%) |
|  | Chest anomalies | Rib anomalies | 6/11 | 1/1 | 2/2 | 4/4 | 1/1 | 1/2 | 2/2 | 1/1 | 2/2 | 20/26 (77%) |
|  |  | Pectus excavatum | 9/11 | - | 1/1 | 3/4 | - | - | - | - | 2/2 | 15/18 (83%) |
|  |  | Pectus carinatum | - | 1/1 | - | - | - | - | - | - | - | 1/1 (100%) |
|  |  | Narrow thorax | - | - | 1/2 | 0/4 | - | - | 1/3 | - | - | 2/9 (22%) |
|  | Limbs and appendages | Long fingers | 6/11 | - | 0/2 | 2/4 | - | - | - | - | 2/2 | 10/19 (53%) |
|  |  | Hyperextensible fingers | 6/11 | 1/1 | 0/2 | 2/4 | - | - | 2/3 | 0/1 | 2/2 | 13/24 (54%) |
|  |  | Clinodactylyl | - | - | - | - | 1/1 | - | - | - | 1/2 | 2/3 (67%) |
|  |  | Genu varus | - | 1/1 | - | - | - | - | - | - | - | 1/1 (100%) |
|  |  | Pes planus | 11/11 | - | 0/1 | 3/4 | 1/1 | 1/2 | - | - | 1/2 | 17/21 (81%) |
|  |  | Talipes equinovarus | 3/11 | - | 0/2 | 1/4 | - | 1/2 | 2/3 | 0/1 | 0/2 | 7/25 (28%) |
|  |  | Generalised joint laxity | - | - | - | - | - | - | 1/3 | - | - | 1/3 (33%) |
|  | Stature | Tall stature (>95%) | 2/11 | - | - | - | - | - | 1/3 | - | 2/2 | 5/16 (31%) |
|  |  | Short stature (<5%) | 3/11 | 1/1 | - | - | 1/1 | - | - | - | - | 5/13 (38%) |
| Neuro-logical | Cerebral imaging | Ventricle abnormalities | 4/7 | - | - | 2/4 | 1/1 | - | 1/3 | - | - | 8/15 (53%) |
|  |  | Corpus callosum dysgenesis/agenesis/hypoplasia | - | 1/1 | 0/1 | 2/4 | 1/1 | 1/2 | 1/3 | - | 2/2 | 8/14 (57%) |
|  |  | Cerebellar abnormalities | - | 1/1 |  |  | - | - | 1/3 | - | - | 2/4 (50%) |
|  |  | Cyst of septum pellucidum | - | - | - | 2/3 | - | - | - | - | - | 2/3 (67%) |
|  |  | Frontotemperal atrophy | - | - | - | - | 1/1 | 1/2 | - | - | - | 2/3 (67%) |
|  |  | Abnormalities in myelination | - | - | - | - | - | 1/2 | - | - | - | 1/2 (50%) |
|  | Other | Unstable gait | 9/9 | - | - | - | - | - | - | - | 1/2 | 10/11 (91%) |
|  |  | Intention tremor | 6/11 | - | - | - | - | - | 2/3 | - | - | 8/14 (57%) |
|  |  | Depressed deep tendon reflexes | 9/10 | - | - | - | - | - | - | - | - | 9/10 (90%) |
|  |  | Epilepsy | - | - | - | - | - |  | - | 1/1 | 2/2 | 3/3 (100%) |
| Psycho-social |  | Intellectual delay | 11/11 | - | - | 4/4 | - | 2/2 | 3/3 | 1/1 | 2/2 | 23/23 (100%) |
|  |  | ASD | - | - | - | - | - | 2/2 | 1/3 | - | - | 3/5 (60%) |
|  |  | Nonverbal | 5/11 | 1/1 | - | 3/4 | - | 1/2 | 1/3 | 0/1 | - | 11/22 (50%) |
|  |  | Speech defect e.g. sluggish speech with hoarse loud voice | 6/6 | - | - | 1/1 | - | - | - | - | 2/2 | 9/9 (100%) |
|  |  | Anxiety | 7/11 | 1/1 | - | - | - | - | - | - | 2/2 | 10/14 (71%) |
|  |  | ADHD | - | - | - | 2/4 | - | - | - | - | - | 2/4 (50%) |
|  |  | Self-mutilation | - | 1/1 | - | 1/4 | - | 1/2 | - | - | - | 3/7 (43%) |
|  |  | Feed themselves | 10/10 | 0/1 | - | 1/4 | - | - | 2/3 | - | - | 13/18 (72%) |
|  |  | Dress themselves | 6/8 | 0/1 | - | 2/4 | - | - | - | - | 1/2 | 9/15 (60%) |
| Psycho-social |  | Toilet training | 6/8 | - | - | 1/4 | - | - | - | 0/1 | 1/2 | 8/15 (53%) |
|  |  | Affable behaviour | - | - | - | 2/3 | - | - | 2/3 | - | - | 4/6 (67%) |
| Other |  | Genital urinary tract anomalies | 5/11 | - | - | - | - | 0/2 | 1/3 | - | 0/2 | 6/18 (33%) |
|  |  | Kidney anomalies | - | - | - | - | 1/1 | - | - | - | - | 1/1 (100%) |
|  |  | Cardiac anomalies | - | - | 0/1 | 2/4 | 0/1 | - | 1/3 | - | 0/2 | 3/11 (27%) |
|  |  | Strabismus | 5/11 | - | - | 1/4 | - | - | - | - | 0/2 | 6/17 (35%) |
|  |  | Optic nerve hypoplasia/atrophy | - | - | - | - | - | 2/2 | - | - | - | 2/2 (100%) |
|  |  | Cryptochordism | - | - | - | 1/1 | - | - | 1/3 | - | - | 2/4 (50%) |
|  |  | Hearing loss | - | - | - | - | - | - | 1/3 | - | - | 1/3 (33%) |
|  |  | Frequent otitis media | 8/11 | - | - | - | - | 1/2 | - | - | - | 9/13 (69%) |
|  |  | Frequent sinusitis | 9/11 | - | - | - | - | 1/2 | - | - | - | 10/13 (77%) |
|  |  | Mild hypertrichosis | 7/11 | - | - | - | - | - | - | - | - | 7/11 (64%) |
|  |  | Dysphagia | - | - | - | - | - | 2/2 | - | - | - | 2/2 (100%) |
|  |  | Constipation | 7/11 | - | - | - | - | - | - | - | - | 7/11 (64%) |
|  |  | Wide spaced nipples | - | - | 2/2 | 3/4 | - | - | - | - | - | 5/6 (83%) |
|  |  | Low body weight (<5%) | 3/11 | 1/1 | - | - | - | - | - | - | - | 4/12 (33%) |
|  |  | Inguinal hernia | - | - | - | - | 1/1 | - | - | - | 0/2 | 1/3 (33%) |
|  |  | (-) not reported |  |  |  |  |  |  |  |  |  |  |

Alanay, Y. *et al.* (2014) ‘TMCO1 deficiency causes autosomal recessive cerebrofaciothoracic dysplasia.’, *American journal of medical genetics. Part A*. United States, 164A(2), pp. 291–304. doi: 10.1002/ajmg.a.36248.

Caglayan, A. O. *et al.* (2013) ‘Whole-exome sequencing identified a patient with TMCO1 defect syndrome and expands the phenotic spectrum.’, *Clinical genetics*, pp. 394–395. doi: 10.1111/cge.12088.

Michael Yates, T., Ng, O. H., *et al.* (2019) ‘Cerebrofaciothoracic dysplasia: Four new patients with a recurrent TMCO1 pathogenic variant’, *American Journal of Medical Genetics, Part A*, 179(1), pp. 43–49. doi: 10.1002/ajmg.a.60678.

Pehlivan, D. *et al.* (2014) ‘Whole-exome sequencing links TMCO1 defect syndrome with cerebro-facio-thoracic dysplasia’, *European journal of human genetics : EJHG*. 2014/01/15. Nature Publishing Group, 22(9), pp. 1145–1148. doi: 10.1038/ejhg.2013.291.

Sharkia, R. *et al.* (2019) ‘A novel biallelic loss-of-function mutation in TMCO1 gene confirming and expanding the phenotype spectrum of cerebro-facio-thoracic dysplasia.’, *American journal of medical genetics. Part A*. United States, 179(7), pp. 1338–1345. doi: 10.1002/ajmg.a.61168.

Tender, J. A. F. and Ferreira, C. R. (2018) ‘Cerebro-facio-thoracic dysplasia (Pascual-Castroviejo syndrome): Identification of a novel mutation, use of facial recognition analysis, and review of the literature’, *Translational science of rare diseases*. IOS Press, 3(1), pp. 37–43. doi: 10.3233/TRD-180022.

Xin, B. *et al.* (2010) ‘Homozygous frameshift mutation in TMCO1 causes a syndrome with craniofacial dysmorphism, skeletal anomalies, and mental retardation.’, *Proceedings of the National Academy of Sciences of the United States of America*, 107(1), pp. 258–263. doi: 10.1073/pnas.0908457107.
